# *N*-[3-(4-Fluoro­benz­yl)-2,4-dioxo-1,3-diaza­spiro­[4.5]dec-8-yl]-2-methyl­benzene­sulfonamide

**DOI:** 10.1107/S160053681105269X

**Published:** 2011-12-14

**Authors:** S. Jeyaseelan, M. Vinduvahini, M. Madaiah, Suman Bhattacharya, H. D. Revanasiddappa

**Affiliations:** a Department of Physics, Yuvaraja’s College (Constituent College), University of Mysore, Mysore 570 005, Karnataka, India; b Department of Physics, Sri D. Devaraja Urs Govt. First Grade College, Hunsur 571 105, Mysore District, Karnataka, India; c Department of Studies in Chemistry, Manasagangotri, University of Mysore, Mysore 570 006, Karnataka, India; d Department of Chemistry, Pondicherry, University, Pondicherry 605 014, India

## Abstract

In the title compound, C_22_H_24_FN_3_O_4_S, the cyclo­hexane ring adopts a chair conformation and the five-membered ring is essentially planar, with a maximum deviation of 0.040 (2) Å. The dihedral angles between the five-membered ring and the tolyl and fluoro­benzene rings are 56.74 (12) and 89.88 (12)°, respectively. The two terminal benzene rings make a dihedral angle of 63.53 (12)°. The crystal structure displays inter­molecular C—H⋯O and N—H⋯O hydrogen bonds. An intra­molecular C—H⋯O hydrogen bond also occurs.

## Related literature

For the biological activity of related compounds, see: Cartwright *et al.* (2007 ▶); Collins (2000 ▶); Warshakoon *et al.* (2006 ▶) and for their pharmaceutical activity, see: Kiselyov *et al.* (2006 ▶); Sakthivel & Cook (2005 ▶); Eldrup *et al.* (2004 ▶); Bamford *et al.* (2005) ▶; Puerstinger *et al.* (2006 ▶).
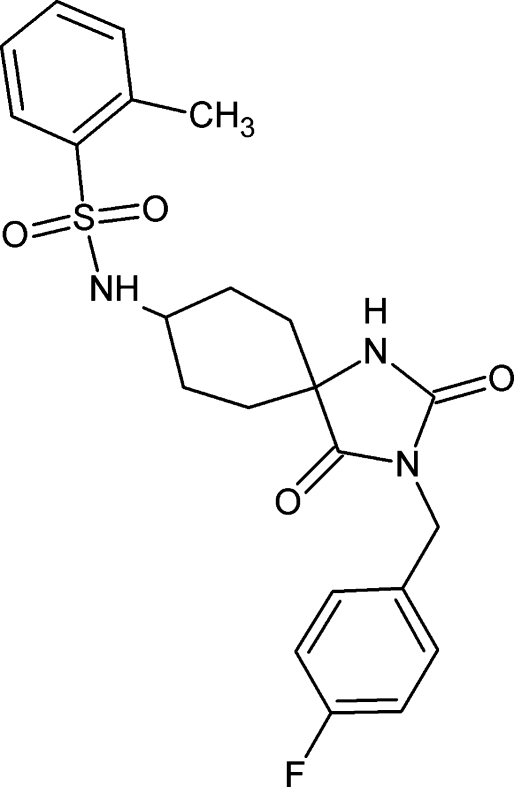


## Experimental

### 

#### Crystal data

C_22_H_24_FN_3_O_4_S
*M* *_r_* = 445.50
Monoclinic, 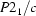

*a* = 5.8314 (3) Å
*b* = 26.3603 (11) Å
*c* = 13.8558 (7) Å
β = 98.623 (5)°
*V* = 2105.80 (18) Å^3^
*Z* = 4
Mo *K*α radiation
μ = 0.20 mm^−1^
*T* = 293 K
0.20 × 0.15 × 0.12 mm

#### Data collection

Oxford Diffraction Xcalibur diffractometer
Absorption correction: multi-scan (*CrysAlis PRO RED*; Oxford Diffraction, 2010 ▶) *T*_min_ = 0.771, *T*_max_ = 1.000
23208 measured reflections
3693 independent reflections
3034 reflections with *I* > 2σ(*I*)
*R*_int_ = 0.044

#### Refinement

*R*[*F* ^2^ > 2σ(*F* ^2^)] = 0.043
*wR*(*F* ^2^) = 0.114
*S* = 1.06
3693 reflections
280 parameters
H-atom parameters constrained
Δρ_max_ = 0.46 e Å^−3^
Δρ_min_ = −0.43 e Å^−3^

### 

Data collection: *CrysAlis PRO CCD* (Oxford Diffraction, 2010 ▶); cell refinement: *CrysAlis PRO CCD*; data reduction: *CrysAlis PRO RED* (Oxford Diffraction, 2010 ▶); program(s) used to solve structure: *SHELXS97* (Sheldrick, 2008 ▶); program(s) used to refine structure: *SHELXL97* (Sheldrick, 2008 ▶); molecular graphics: *ORTEP-3* (Farrugia, 1997 ▶) and *CAMERON* (Watkin *et al.*, 1993 ▶); software used to prepare material for publication: *WinGX* (Farrugia, 1999 ▶).

## Supplementary Material

Crystal structure: contains datablock(s) I, global. DOI: 10.1107/S160053681105269X/wn2460sup1.cif

Structure factors: contains datablock(s) I. DOI: 10.1107/S160053681105269X/wn2460Isup2.hkl

Supplementary material file. DOI: 10.1107/S160053681105269X/wn2460Isup3.cml

Additional supplementary materials:  crystallographic information; 3D view; checkCIF report

## Figures and Tables

**Table 1:** Hydrogen-bond geometry (Å, °)

*D*—H⋯*A*	*D*—H	H⋯*A*	*D*⋯*A*	*D*—H⋯*A*
N7—H7⋯O4^i^	0.86	2.04	2.885 (2)	166
N9—H9⋯O6^ii^	0.86	2.24	3.013 (2)	149
C12—H12⋯O3^iii^	0.93	2.59	3.290 (3)	132
C31—H31*A*⋯O6	0.96	2.20	2.973 (3)	137
C31—H31*C*⋯O5^iv^	0.96	2.47	3.238 (3)	137

Symmetry codes: (i) 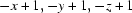; (ii) 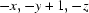; (iii) 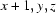; (iv) 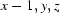.

**Fig. 1.**
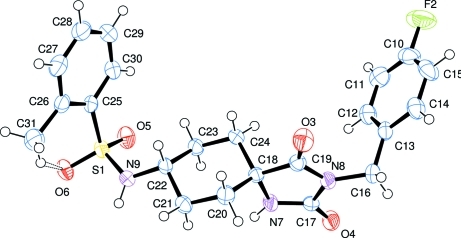
The molecular structure with displacement ellipsoids drawn at the 50% probability level. The H atoms are shown as spheres of arbitrary radius. The dashed lines indicate the intramolecular hydrogen bond.

**Fig. 2.**
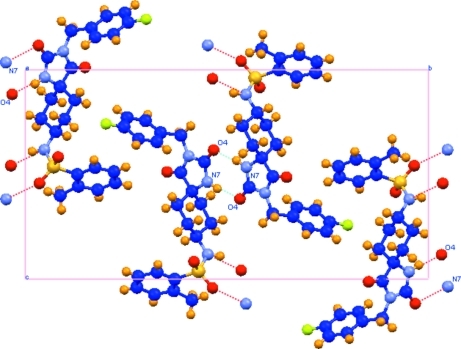
A view of the crystal structure down the a axis. Dashed lines indicate hydrogen bonds.

## supplementary crystallographic information

### Comment

One of the challenges of medicinal chemistry is the promotion of structural diversity,which can be achieved by the attachment of pharmacophoric groups to significant molecular scaffolds in combinatorial chemistry. An example of such a process includes *di*- and *tri*-substituted hydantoins, which have been widely used in biological screenings, resulting in numerous pharmaceutical applications (Cartwright *et al.*, 2007; Collins, 2000; Warshakoon *et al.*, 2006). Hydantoin analogues have shown versatile therapeutic applications and some of them have been approved as drugs. For example, fosphenytoin, as a sodium channel antagonist, is used for the treatment of epilepsy. Phenytoin has antiarrhythmic, anticonvulsant and antineuralgic activities. Ethotoin and mephenytoin both show anticonvulsant activity. Nilutamide is used in the treatment of prostate cancer (Kiselyov *et al.*, 2006; Sakthivel & Cook, 2005; Eldrup *et al.*, 2004; Bamford *et al.*, 2005; Puerstinger *et al.*, 2006).

The asymmetric unit of *N*-[3-(4-fluorobenzyl)-2,4-dioxo- 1,3-diazaspiro[4.5] dec-8-yl]-2-methylbenzenesulfonamide, C_22_H_24_FN_3_O_4_S, contains just one molecule (Fig. 1). The cyclohexane ring adopts a chair conformation and the five-membered ring is essentially planar, with a maximum deviation from planarity of 0.040 (2) Å, for atom C18. The dihedral angles between the five-membered ring and the (C25—C30) and (C10—C15) rings are 56.74 (12)° and 89.88 (12)°, respectively. The two terminal aromatic rings make a dihedral angle of 63.53 (12)°. The crystal structure displays intermolecular N7—H7···O4, N9—H9···O6, C12—H12···O3 and C31—H31C···O5 hydrogen bonds (Table 1); an intramolecular C31—H31A···O6 hydrogen bond is also observed. The packing of the molecules in the title structure is depicted in Fig. 2. A short contact of 2.04 Å was observed for H9···H31A.

### Experimental

A mixture of *tert*-butyl (4-oxocyclohexyl)carbamate (2 g, 9.37 mmol) and ammonium carbonate (1.08 g, 11.2 mmol) were taken up in ethanol and water, respectively. A solution of sodium cyanide (2 g, 9.37 mmol) in water (6 ml) was added dropwise and the reaction mixture was stirred at RT for 24 hrs. A mixture of anhydrous potassium carbonate (1.28 g, 9.31 mmol) and 1-(bromomethyl)-4-fluorobenzene (1.30 g, 6.9 mmol) in DMF (20ml) was refluxed and the solid was filtered, washed with water and dried in vacuum to yield hydantoin. The *tert*-butyl dicarbonate (BOC) was de-protected using dioxane-HCl and rendered basic to obtain the free amine. A mixture of the product (0.2 g, 0.686 mmol), triethylamine (0.083 g, 0.82 mmol) and sulfonyl  chloride (0.083 g, 0.617 mmol) in dichloromethane (10ml) was stirred at room temperature. After completion of the reaction (checked by TLC) the product was concentrated in vacuum to give the title compound (160 mg, 53%), which was recrystalized using 1:1 hexane: ethyl acetate as solvent.

### Refinement

All H atoms were placed at calculated positions and refined as riding, with N—H = 0.86 Å, C(methine)—H = 0.98 Å, C(methylene)—H = 0.97 Å , Csp^2^—H = 0.93 Å and C(methyl)—H = 0.96 Å. *U*_iso_(H) = x*U*_eq_(C,N), where x = 1.5 for methyl H and 1.2 for all other H atoms.

### Crystal data

**Table:**

C_22_H_24_FN_3_O_4_S	*F*(000) = 936
*M**_r_* = 445.50	*D*_x_ = 1.405 Mg m^−^^3^
Monoclinic, *P*2_1_/*c*	Melting point: 425 K
Hall symbol: -P 2ybc	Mo *K*α radiation, λ = 0.71073 Å
*a* = 5.8314 (3) Å	Cell parameters from 3693 reflections
*b* = 26.3603 (11) Å	θ = 2.8–25.0°
*c* = 13.8558 (7) Å	µ = 0.20 mm^−^^1^
β = 98.623 (5)°	*T* = 293 K
*V* = 2105.80 (18) Å^3^	Prism, colourless
*Z* = 4	0.20 × 0.15 × 0.12 mm

### Data collection

**Table:**

Oxford Diffraction Xcalibur diffractometer	3693 independent reflections
Radiation source: fine-focus sealed tube	3034 reflections with *I* > 2σ(*I*)
graphite	*R*_int_ = 0.044
Detector resolution: 15.9821 pixels mm^-1^	θ_max_ = 25.0°, θ_min_ = 2.8°
ω scans	*h* = −6→6
Absorption correction: multi-scan (*CrysAlis PRO RED*; Oxford Diffraction, 2010)	*k* = −31→31
*T*_min_ = 0.771, *T*_max_ = 1.000	*l* = −16→16
23208 measured reflections	

### Refinement

**Table:**

Refinement on *F*^2^	Primary atom site location: structure-invariant direct methods
Least-squares matrix: full	Secondary atom site location: difference Fourier map
*R*[*F*^2^ > 2σ(*F*^2^)] = 0.043	Hydrogen site location: inferred from neighbouring sites
*wR*(*F*^2^) = 0.114	H-atom parameters constrained
*S* = 1.06	*w* = 1/[σ^2^(*F*_o_^2^) + (0.0528*P*)^2^ + 1.3845*P*] where *P* = (*F*_o_^2^ + 2*F*_c_^2^)/3
3693 reflections	(Δ/σ)_max_ = 0.001
280 parameters	Δρ_max_ = 0.46 e Å^−^^3^
0 restraints	Δρ_min_ = −0.43 e Å^−^^3^

### Special details

**Table:**

Experimental. *CrysAlis PRO*, Oxford Diffraction Ltd., Version 1.171.33.55 (release 05–01–2010 CrysAlis171. NET) Empirical absorption correction using spherical harmonics, implemented in SCALE3 ABSPACK scaling algorithm.Colourless solid; Yield: 98 mg, 64%; mp:425 K; IR cm^-1^ (KBr) 3355 (N—H), 1652, 1344 (S=O); Anal.Calcd for C_22_H_24_FN_3_O_4_S: C, 59.31; H, 5.43; N, 9.43%, Found, C, 58.75; H, 5.39; N, 9.11%.
Geometry. All s.u.'s (except the s.u. in the dihedral angle between two l.s. planes) are estimated using the full covariance matrix. The cell s.u.'s are taken into account individually in the estimation of s.u.'s in distances, angles and torsion angles; correlations between s.u.'s in cell parameters are only used when they are defined by crystal symmetry. An approximate (isotropic) treatment of cell s.u.'s is used for estimating s.u.'s involving l.s. planes.
Refinement. Refinement of *F*^2^ against ALL reflections. The weighted *R*-factor *wR* and goodness of fit *S* are based on *F*^2^, conventional *R*-factors *R* are based on *F*, with *F* set to zero for negative *F*^2^. The threshold expression of *F*^2^ > 2σ(*F*^2^) is used only for calculating *R*-factors(gt) *etc*. and is not relevant to the choice of reflections for refinement. *R*-factors based on *F*^2^ are statistically about twice as large as those based on *F*, and *R*-factors based on ALL data will be even larger.

### Fractional atomic coordinates and isotropic or equivalent isotropic displacement parameters (Å^2^)

**Table:**

	*x*	*y*	*z*	*U*_iso_*/*U*_eq_	
S1	0.25585 (10)	0.57039 (2)	0.03784 (4)	0.03284 (17)	
F2	0.6870 (4)	0.79980 (6)	0.74198 (16)	0.0846 (6)	
O3	−0.0805 (3)	0.64828 (7)	0.51242 (13)	0.0477 (5)	
O4	0.4811 (3)	0.53253 (6)	0.61681 (12)	0.0437 (4)	
O5	0.4857 (3)	0.58150 (7)	0.08365 (12)	0.0435 (4)	
O6	0.2245 (3)	0.53545 (6)	−0.04209 (12)	0.0439 (4)	
N7	0.2751 (3)	0.54833 (7)	0.46471 (13)	0.0342 (4)	
H7	0.3265	0.5239	0.4328	0.041*	
N8	0.1988 (3)	0.59465 (7)	0.58837 (13)	0.0320 (4)	
N9	0.1103 (3)	0.54742 (7)	0.11805 (13)	0.0342 (4)	
H9	0.0578	0.5170	0.1098	0.041*	
C10	0.5716 (5)	0.75487 (9)	0.7276 (2)	0.0512 (7)	
C11	0.6523 (5)	0.71902 (10)	0.6701 (2)	0.0474 (6)	
H11	0.7829	0.7251	0.6406	0.057*	
C12	0.5351 (4)	0.67352 (9)	0.65691 (17)	0.0396 (6)	
H12	0.5881	0.6486	0.6182	0.048*	
C13	0.3403 (4)	0.66429 (8)	0.70011 (15)	0.0315 (5)	
C14	0.2645 (4)	0.70179 (9)	0.75752 (17)	0.0407 (6)	
H14	0.1335	0.6962	0.7869	0.049*	
C15	0.3800 (5)	0.74727 (10)	0.7718 (2)	0.0509 (7)	
H15	0.3289	0.7723	0.8107	0.061*	
C16	0.2155 (4)	0.61414 (9)	0.68760 (16)	0.0369 (5)	
H16A	0.2971	0.5897	0.7325	0.044*	
H16B	0.0606	0.6182	0.7040	0.044*	
C17	0.3364 (4)	0.55541 (8)	0.56046 (16)	0.0312 (5)	
C18	0.1119 (3)	0.58646 (8)	0.41997 (15)	0.0267 (4)	
C19	0.0576 (4)	0.61430 (8)	0.50965 (16)	0.0309 (5)	
C20	−0.1057 (4)	0.56436 (9)	0.36050 (17)	0.0368 (5)	
H20A	−0.2186	0.5912	0.3442	0.044*	
H20B	−0.1728	0.5395	0.3998	0.044*	
C21	−0.0561 (4)	0.53917 (9)	0.26671 (16)	0.0347 (5)	
H21A	0.0427	0.5098	0.2830	0.042*	
H21B	−0.2005	0.5275	0.2294	0.042*	
C22	0.0622 (4)	0.57566 (8)	0.20473 (15)	0.0293 (5)	
H22	−0.0443	0.6036	0.1835	0.035*	
C23	0.2805 (4)	0.59716 (9)	0.26402 (16)	0.0335 (5)	
H23A	0.3504	0.6215	0.2248	0.040*	
H23B	0.3912	0.5700	0.2815	0.040*	
C24	0.2269 (4)	0.62287 (8)	0.35617 (15)	0.0322 (5)	
H24A	0.1248	0.6515	0.3384	0.039*	
H24B	0.3696	0.6357	0.3931	0.039*	
C25	0.1245 (4)	0.62927 (8)	0.00021 (15)	0.0316 (5)	
C26	−0.0956 (4)	0.63160 (9)	−0.05589 (16)	0.0350 (5)	
C27	−0.1911 (5)	0.67963 (10)	−0.07325 (18)	0.0448 (6)	
H27	−0.3385	0.6825	−0.1093	0.054*	
C28	−0.0752 (5)	0.72314 (10)	−0.03893 (19)	0.0517 (7)	
H28	−0.1452	0.7546	−0.0516	0.062*	
C29	0.1422 (5)	0.72009 (10)	0.01356 (19)	0.0523 (7)	
H29	0.2219	0.7494	0.0357	0.063*	
C30	0.2426 (5)	0.67305 (9)	0.03346 (17)	0.0418 (6)	
H30	0.3904	0.6707	0.0694	0.050*	
C31	−0.2300 (4)	0.58652 (10)	−0.0997 (2)	0.0476 (6)	
H31A	−0.1433	0.5561	−0.0817	0.071*	
H31B	−0.2565	0.5897	−0.1695	0.071*	
H31C	−0.3761	0.5849	−0.0759	0.071*	

### Atomic displacement parameters (Å^2^)

**Table:**

	*U*^11^	*U*^22^	*U*^33^	*U*^12^	*U*^13^	*U*^23^
S1	0.0350 (3)	0.0353 (3)	0.0273 (3)	0.0006 (2)	0.0020 (2)	−0.0056 (2)
F2	0.0989 (15)	0.0430 (9)	0.1017 (15)	−0.0217 (9)	−0.0184 (12)	0.0032 (10)
O3	0.0504 (10)	0.0498 (10)	0.0435 (10)	0.0222 (9)	0.0094 (8)	−0.0031 (8)
O4	0.0563 (11)	0.0373 (9)	0.0333 (9)	0.0142 (8)	−0.0068 (8)	−0.0005 (7)
O5	0.0328 (9)	0.0554 (10)	0.0404 (9)	0.0026 (8)	−0.0009 (7)	−0.0069 (8)
O6	0.0513 (10)	0.0443 (10)	0.0363 (9)	0.0004 (8)	0.0074 (8)	−0.0140 (8)
N7	0.0474 (12)	0.0268 (9)	0.0269 (9)	0.0127 (8)	0.0007 (8)	−0.0041 (8)
N8	0.0391 (11)	0.0300 (9)	0.0267 (9)	0.0038 (8)	0.0045 (8)	−0.0027 (8)
N9	0.0451 (11)	0.0272 (9)	0.0289 (10)	−0.0047 (8)	0.0013 (8)	−0.0027 (8)
C10	0.0607 (18)	0.0321 (13)	0.0532 (16)	−0.0021 (12)	−0.0164 (14)	0.0063 (12)
C11	0.0402 (14)	0.0541 (16)	0.0460 (15)	−0.0047 (12)	−0.0002 (11)	0.0069 (13)
C12	0.0404 (13)	0.0448 (14)	0.0347 (12)	0.0041 (11)	0.0091 (10)	−0.0055 (11)
C13	0.0348 (12)	0.0347 (12)	0.0240 (10)	0.0071 (9)	0.0014 (9)	−0.0026 (9)
C14	0.0397 (13)	0.0448 (14)	0.0371 (13)	0.0119 (11)	0.0043 (11)	−0.0068 (11)
C15	0.0618 (18)	0.0360 (14)	0.0508 (16)	0.0161 (12)	−0.0048 (14)	−0.0124 (12)
C16	0.0459 (14)	0.0399 (13)	0.0261 (11)	0.0004 (10)	0.0094 (10)	−0.0034 (10)
C17	0.0399 (13)	0.0227 (10)	0.0300 (11)	0.0024 (9)	0.0020 (10)	0.0003 (9)
C18	0.0282 (11)	0.0240 (10)	0.0272 (11)	0.0029 (8)	0.0016 (9)	−0.0003 (9)
C19	0.0320 (12)	0.0296 (11)	0.0317 (12)	0.0002 (9)	0.0067 (9)	0.0000 (9)
C20	0.0323 (12)	0.0442 (13)	0.0343 (12)	−0.0093 (10)	0.0059 (10)	−0.0010 (10)
C21	0.0322 (12)	0.0368 (12)	0.0333 (12)	−0.0101 (10)	−0.0011 (9)	−0.0042 (10)
C22	0.0329 (12)	0.0281 (11)	0.0248 (11)	0.0012 (9)	−0.0023 (9)	−0.0020 (9)
C23	0.0356 (12)	0.0358 (12)	0.0292 (11)	−0.0105 (10)	0.0047 (9)	−0.0024 (10)
C24	0.0386 (12)	0.0277 (11)	0.0296 (11)	−0.0071 (9)	0.0022 (10)	−0.0020 (9)
C25	0.0395 (13)	0.0344 (12)	0.0214 (10)	−0.0026 (10)	0.0064 (9)	−0.0016 (9)
C26	0.0389 (13)	0.0413 (13)	0.0249 (11)	−0.0021 (10)	0.0054 (10)	0.0034 (10)
C27	0.0470 (15)	0.0514 (15)	0.0348 (13)	0.0073 (12)	0.0018 (11)	0.0068 (12)
C28	0.077 (2)	0.0395 (14)	0.0373 (14)	0.0141 (14)	0.0059 (14)	0.0038 (12)
C29	0.080 (2)	0.0354 (14)	0.0393 (14)	−0.0076 (13)	0.0014 (14)	−0.0058 (11)
C30	0.0506 (15)	0.0419 (14)	0.0307 (12)	−0.0073 (11)	−0.0008 (11)	−0.0038 (11)
C31	0.0400 (14)	0.0532 (15)	0.0462 (15)	−0.0072 (12)	−0.0047 (11)	0.0029 (12)

### Geometric parameters (Å, °)

**Table:**

S1—O5	1.4248 (17)	C18—C20	1.522 (3)
S1—O6	1.4310 (16)	C18—C24	1.526 (3)
S1—N9	1.6141 (19)	C20—C21	1.525 (3)
S1—C25	1.774 (2)	C20—H20A	0.9700
F2—C10	1.362 (3)	C20—H20B	0.9700
O3—C19	1.209 (3)	C21—C22	1.522 (3)
O4—C17	1.219 (3)	C21—H21A	0.9700
N7—C17	1.334 (3)	C21—H21B	0.9700
N7—C18	1.457 (3)	C22—C23	1.518 (3)
N7—H7	0.8600	C22—H22	0.9800
N8—C19	1.366 (3)	C23—C24	1.519 (3)
N8—C17	1.399 (3)	C23—H23A	0.9700
N8—C16	1.457 (3)	C23—H23B	0.9700
N9—C22	1.475 (3)	C24—H24A	0.9700
N9—H9	0.8600	C24—H24B	0.9700
C10—C11	1.364 (4)	C25—C30	1.387 (3)
C10—C15	1.367 (4)	C25—C26	1.399 (3)
C11—C12	1.379 (4)	C26—C27	1.390 (3)
C11—H11	0.9300	C26—C31	1.501 (3)
C12—C13	1.383 (3)	C27—C28	1.379 (4)
C12—H12	0.9300	C27—H27	0.9300
C13—C14	1.382 (3)	C28—C29	1.366 (4)
C13—C16	1.507 (3)	C28—H28	0.9300
C14—C15	1.375 (4)	C29—C30	1.381 (4)
C14—H14	0.9300	C29—H29	0.9300
C15—H15	0.9300	C30—H30	0.9300
C16—H16A	0.9700	C31—H31A	0.9600
C16—H16B	0.9700	C31—H31B	0.9600
C18—C19	1.517 (3)	C31—H31C	0.9600
			
O5—S1—O6	118.65 (10)	C21—C20—H20A	109.2
O5—S1—N9	109.09 (10)	C18—C20—H20B	109.2
O6—S1—N9	105.79 (10)	C21—C20—H20B	109.2
O5—S1—C25	106.68 (11)	H20A—C20—H20B	107.9
O6—S1—C25	109.65 (10)	C22—C21—C20	111.44 (18)
N9—S1—C25	106.39 (10)	C22—C21—H21A	109.3
C17—N7—C18	112.76 (17)	C20—C21—H21A	109.3
C17—N7—H7	123.6	C22—C21—H21B	109.3
C18—N7—H7	123.6	C20—C21—H21B	109.3
C19—N8—C17	111.21 (18)	H21A—C21—H21B	108.0
C19—N8—C16	124.50 (18)	N9—C22—C23	112.51 (18)
C17—N8—C16	124.18 (18)	N9—C22—C21	107.33 (17)
C22—N9—S1	123.70 (15)	C23—C22—C21	110.18 (17)
C22—N9—H9	118.1	N9—C22—H22	108.9
S1—N9—H9	118.1	C23—C22—H22	108.9
F2—C10—C11	118.8 (3)	C21—C22—H22	108.9
F2—C10—C15	118.7 (3)	C22—C23—C24	111.09 (18)
C11—C10—C15	122.5 (2)	C22—C23—H23A	109.4
C10—C11—C12	118.2 (3)	C24—C23—H23A	109.4
C10—C11—H11	120.9	C22—C23—H23B	109.4
C12—C11—H11	120.9	C24—C23—H23B	109.4
C11—C12—C13	121.2 (2)	H23A—C23—H23B	108.0
C11—C12—H12	119.4	C23—C24—C18	111.72 (17)
C13—C12—H12	119.4	C23—C24—H24A	109.3
C14—C13—C12	118.5 (2)	C18—C24—H24A	109.3
C14—C13—C16	120.2 (2)	C23—C24—H24B	109.3
C12—C13—C16	121.2 (2)	C18—C24—H24B	109.3
C15—C14—C13	121.0 (2)	H24A—C24—H24B	107.9
C15—C14—H14	119.5	C30—C25—C26	121.1 (2)
C13—C14—H14	119.5	C30—C25—S1	117.35 (18)
C10—C15—C14	118.6 (2)	C26—C25—S1	121.43 (17)
C10—C15—H15	120.7	C27—C26—C25	116.5 (2)
C14—C15—H15	120.7	C27—C26—C31	118.7 (2)
N8—C16—C13	112.37 (18)	C25—C26—C31	124.7 (2)
N8—C16—H16A	109.1	C28—C27—C26	122.3 (2)
C13—C16—H16A	109.1	C28—C27—H27	118.8
N8—C16—H16B	109.1	C26—C27—H27	118.8
C13—C16—H16B	109.1	C29—C28—C27	120.2 (2)
H16A—C16—H16B	107.9	C29—C28—H28	119.9
O4—C17—N7	128.6 (2)	C27—C28—H28	119.9
O4—C17—N8	124.0 (2)	C28—C29—C30	119.4 (2)
N7—C17—N8	107.40 (18)	C28—C29—H29	120.3
N7—C18—C19	100.86 (16)	C30—C29—H29	120.3
N7—C18—C20	113.88 (18)	C29—C30—C25	120.4 (2)
C19—C18—C20	111.67 (17)	C29—C30—H30	119.8
N7—C18—C24	111.37 (18)	C25—C30—H30	119.8
C19—C18—C24	109.52 (17)	C26—C31—H31A	109.5
C20—C18—C24	109.28 (17)	C26—C31—H31B	109.5
O3—C19—N8	125.5 (2)	H31A—C31—H31B	109.5
O3—C19—C18	127.3 (2)	C26—C31—H31C	109.5
N8—C19—C18	107.20 (17)	H31A—C31—H31C	109.5
C18—C20—C21	112.24 (18)	H31B—C31—H31C	109.5
C18—C20—H20A	109.2		
			
O5—S1—N9—C22	62.17 (19)	C20—C18—C19—N8	126.81 (19)
O6—S1—N9—C22	−169.14 (16)	C24—C18—C19—N8	−112.00 (19)
C25—S1—N9—C22	−52.56 (19)	N7—C18—C20—C21	−70.2 (2)
F2—C10—C11—C12	179.4 (2)	C19—C18—C20—C21	176.36 (18)
C15—C10—C11—C12	−0.2 (4)	C24—C18—C20—C21	55.0 (2)
C10—C11—C12—C13	0.3 (4)	C18—C20—C21—C22	−55.6 (3)
C11—C12—C13—C14	−0.2 (4)	S1—N9—C22—C23	−52.8 (2)
C11—C12—C13—C16	−178.6 (2)	S1—N9—C22—C21	−174.14 (15)
C12—C13—C14—C15	−0.1 (3)	C20—C21—C22—N9	178.00 (17)
C16—C13—C14—C15	178.3 (2)	C20—C21—C22—C23	55.2 (2)
F2—C10—C15—C14	−179.7 (2)	N9—C22—C23—C24	−176.14 (17)
C11—C10—C15—C14	−0.1 (4)	C21—C22—C23—C24	−56.4 (2)
C13—C14—C15—C10	0.3 (4)	C22—C23—C24—C18	57.9 (2)
C19—N8—C16—C13	−72.2 (3)	N7—C18—C24—C23	70.5 (2)
C17—N8—C16—C13	103.6 (2)	C19—C18—C24—C23	−178.80 (18)
C14—C13—C16—N8	141.1 (2)	C20—C18—C24—C23	−56.2 (2)
C12—C13—C16—N8	−40.5 (3)	O5—S1—C25—C30	−10.0 (2)
C18—N7—C17—O4	−174.2 (2)	O6—S1—C25—C30	−139.68 (18)
C18—N7—C17—N8	7.0 (3)	N9—S1—C25—C30	106.36 (19)
C19—N8—C17—O4	178.1 (2)	O5—S1—C25—C26	173.77 (18)
C16—N8—C17—O4	1.9 (3)	O6—S1—C25—C26	44.1 (2)
C19—N8—C17—N7	−3.0 (3)	N9—S1—C25—C26	−69.9 (2)
C16—N8—C17—N7	−179.2 (2)	C30—C25—C26—C27	−2.1 (3)
C17—N7—C18—C19	−7.7 (2)	S1—C25—C26—C27	173.96 (18)
C17—N7—C18—C20	−127.5 (2)	C30—C25—C26—C31	176.3 (2)
C17—N7—C18—C24	108.4 (2)	S1—C25—C26—C31	−7.6 (3)
C17—N8—C19—O3	179.4 (2)	C25—C26—C27—C28	1.1 (4)
C16—N8—C19—O3	−4.3 (4)	C31—C26—C27—C28	−177.4 (2)
C17—N8—C19—C18	−1.9 (2)	C26—C27—C28—C29	0.5 (4)
C16—N8—C19—C18	174.32 (19)	C27—C28—C29—C30	−1.2 (4)
N7—C18—C19—O3	−175.9 (2)	C28—C29—C30—C25	0.3 (4)
C20—C18—C19—O3	−54.6 (3)	C26—C25—C30—C29	1.5 (4)
C24—C18—C19—O3	66.6 (3)	S1—C25—C30—C29	−174.8 (2)
N7—C18—C19—N8	5.5 (2)		

### Hydrogen-bond geometry (Å, °)

**Table:**

*D*—H···*A*	*D*—H	H···*A*	*D*···*A*	*D*—H···*A*
N7—H7···O4^i^	0.86	2.04	2.885 (2)	166.
N9—H9···O6^ii^	0.86	2.24	3.013 (2)	149.
C12—H12···O3^iii^	0.93	2.59	3.290 (3)	132.
C31—H31A···O6	0.96	2.20	2.973 (3)	137.
C31—H31C···O5^iv^	0.96	2.47	3.238 (3)	137.

Symmetry codes: (i) −*x*+1, −*y*+1, −*z*+1; (ii) −*x*, −*y*+1, −*z*; (iii) *x*+1, *y*, *z*; (iv) *x*−1, *y*, *z*.
